# MLH1 mediates PARP-dependent cell death in response to the methylating agent *N*-methyl-*N*-nitrosourea

**DOI:** 10.1038/sj.bjc.6605186

**Published:** 2009-07-21

**Authors:** J R McDaid, J Loughery, P Dunne, J C Boyer, C S Downes, R A Farber, C P Walsh

**Affiliations:** 1 Stem Cells and Epigenetics Research Group, Centre for Molecular Biosciences, School of Biomedical Sciences, University of Ulster, Coleraine BT52 1SA, Northern Ireland, UK; 2 Department of Pathology and Laboratory Medicine, University of North Carolina at Chapel Hill, Chapel Hill, NC 27599, USA; 3 Cancer and Ageing Research Group, Centre for Molecular Biosciences, School of Biomedical Sciences, University of Ulster, Coleraine BT52 1SA, Northern Ireland, UK

**Keywords:** alkylating agent, caspase, p53, ATM/ATR

## Abstract

**Background:**

Methylating agents such as *N*-methyl-*N*-nitrosourea (MNU) can cause cell cycle arrest and death either via caspase-dependent apoptosis or via a poly(ADP-ribose) polymerase (PARP)-dependent form of apoptosis. We wished to investigate the possible role of MLH1 in signalling cell death through PARP.

**Methods:**

Fibroblasts are particularly dependent on a PARP-mediated cell death response to methylating agents. We used hTERT-immortalised normal human fibroblasts (WT) to generate isogenic MLH1-depleted cells, confirmed by quantitative PCR and western blotting. Drug resistance was measured by clonogenic and cell viability assays and effects on the cell cycle by cell sorting. Damage signalling was additionally investigated using immunostaining.

**Results:**

MLH1-depleted cells were more resistant to MNU, as expected. Despite having an intact G_2_/M checkpoint, the WT cells did not initially undergo cell cycle arrest but instead triggered cell death directly by PARP overactivation and nuclear translocation of apoptosis-inducing factor (AIF). The MLH1-depleted cells showed defects in this pathway, with decreased staining for phosphorylated H2AX, altered PARP activity and reduced AIF translocation. Inhibitors of PARP, but not of caspases, blocked AIF translocation and greatly decreased short-term cell death in both WT and MLH1-depleted cells. This MLH1-dependent response to MNU was not blocked by inhibitors of ATM/ATR or p53.

**Conclusion:**

These novel data indicate an important role for MLH1 in signalling PARP-dependent cell death in response to the methylating agent MNU.

Mismatch repair (MMR) is an essential system for reducing postreplicative errors in DNA including base–base mispairs and small insertion/deletion loops due to polymerase slippage, and is conserved from bacteria to humans. In *E. coli* it consists of two key homodimeric proteins: MutS, which recognises and binds the mismatch, and MutL, which is recruited to the complex and initiates repair (Kunkel and Erie, 2005). In humans there are multiple homologues for each protein and they combine to form alternative repair complexes with slightly different specificities: MutS*α*, a heterodimer of MSH2 and MSH6, can bind single base mismatches or insertion/deletion loops, whereas MutS*β*, a heterodimer of MSH2 and MSH3 only binds to insertion/deletion loops (Kunkel and Erie, 2005). For somatic cells, the primary MutL complex is called MutL*α* and consists of a heterodimer of MLH1 and PMS2, which binds to MutS and initiates repair (Raschle *et al*, 2002). Excision of the damaged base and resynthesis involves participation of other proteins such as PCNA, EXO1 and DNA polymerases *δ* and *ε* (Kunkel and Erie, 2005). In the absence of functional MMR, high rates of mutation are seen, particularly at microsatellite repeats, which can lead to inactivating frameshifts in mononucleotide runs within the coding regions of genes such as *BLM*, *BAX* and *IGFIIR*, disrupting their function (Duval and Hamelin, 2002). This mutator phenotype also complicates the separation of primary and secondary effects of MLH1-deficiency.

Mutations or epigenetic silencing of MMR genes is associated with several human cancers. Lynch Syndrome, or hereditary non-polyposis colorectal cancer, arises as a result of a primary defect in MMR genes, most commonly *MLH1* and *MSH2* (Peltomaki and Vasen, 2004); patients have a very high risk of developing colorectal and/or endometrial tumours and are at elevated risk for certain other types of tumours. Defects in MMR are also found in sporadic cancers of the colon, stomach, endometrium and ovary (Thibodeau *et al*, 1998). For most sporadic cancers, inactivation of MLH1 is usually associated with methylation of the promoter rather than mutation (Herman and Baylin, 2003). MMR-defective tumours can also arise in response to exposure to some cytotoxic agents, with microsatellite instability being reported in lung cancer in chromium-exposed workers (Hirose *et al*, 2002) and in glioblastomas after temozolomide treatment (Cahill *et al*, 2007).

MMR proteins also participate in DNA damage signalling and MMR-deficient cells show resistance to a number of different classes of chemotherapeutic drugs (reviewed in O'Brien and Brown, 2006) such as the methylating agent *N*-methyl-*N*-nitrosourea (MNU), the cross-linking agent cisplatin and the antimetabolite 6-thioguanine (6TG). MLH1 has been shown to participate in signalling cell cycle arrest in response to 6TG and MNU, or to the related methylating agent *N*-methyl-*N′*-nitro-*N*-nitrosoguanidine (MNNG; Hawn *et al*, 1995; Buermeyer *et al*, 1999). For MNNG, MLH1 signals arrest in conjunction with the phosphatidylinositol 3-kinase-like kinases Ataxia telangiectasia mutated (ATM; Adamson *et al*, 2005) and ATM- and Rad3-related (ATR; Stojic *et al*, 2004). These kinases phosphorylate a number of targets in the cell in response to damage, such as the variant histone H2AX and p53 (Lavin *et al*, 2005). Treatment of cells with MNNG (Yanamadala and Ljungman, 2003) also leads to stabilisation of p53 and subsequently triggers apoptosis through a caspase-mediated pathway, which is dependent on MLH1 (Hickman and Samson, 1999, 2004; Yanamadala and Ljungman, 2003). This pathway is primarily important in cells which are deficient in methylguanine methyltransferase (MGMT), indicating that the main cytotoxic lesion caused by the methylating agents is 0^6^-methylguanine (Hickman and Samson, 2004). More recently, MLH1 has been shown by Kinsella and co-workers to be important for signalling autophagy and inhibiting apoptosis in response to 6TG, a pathway that also requires p53 (Zeng *et al*, 2007). The damage signalling function of MLH1 has been shown to require higher levels of the protein than those needed to successfully maintain microsatellite stability, as cells with low levels of MLH1 show no increase in mutation rate but have lost the ability to signal cell cycle arrest in response to 6TG (Buermeyer *et al*, 1999) or MNNG (Buermeyer *et al*, 1999; Cejka *et al*, 2003; Stojic *et al*, 2004).

Methylating agents are in widespread use in chemotherapy, in part, due to their ability to kill apoptosis-resistant cells such as follicular lymphoma (Lister, 1991; Zong *et al*, 2004; Amaravadi and Thompson, 2007). Cells that express the oncogene *bcl-2*, a dominant-negative regulator of apoptosis, or which are deficient in the cellular homologues *BAK* or *BAX*, are resistant to apoptosis through caspase-mediated pathways (Danial, 2007). Methylating agents can trigger an alternative cell death pathway in *Bax*/*Bak* double mutant mouse fibroblast cells (Zong *et al*, 2004; Moubarak *et al*, 2007), or in cells overexpressing *bcl-2* (Amaravadi and Thompson, 2007), involving poly(ADP-ribose) polymerase (PARP). PARP is a nuclear enzyme, which responds to DNA damage by adding 50–200 molecules of ADP-ribose to a variety of nuclear targets, including histones (Kim *et al*, 2005). PARP activation causes a rapid drop in nicotinamide adenine dinucleotide (NAD+) levels, triggering mitochondrial membrane depolarisation and translocation of apoptosis inducing factor (AIF) to the nucleus (Yu *et al*, 2002, 2006). In the nucleus, AIF causes chromatin condensation and DNA fragmentation (Susin *et al*, 1999). However, little is known about the upstream signalling events which activate PARP in response to methylating damage.

Here we sought to address the possible role of MLH1 in caspase-independent programmed cell death in response to methylating agents. We generated an isogenic system of normal human fibroblasts and derivatives, which are MLH1 depleted and resistant to MNU, but lack complications such as microsatellite instability and aneuploidy. We show here that the WT cells initially die almost exclusively by PARP-mediated cell death, and that this pathway is inhibited in cells with decreased levels of MLH1. These data uncover a new role for MLH1 in regulating a caspase-independent, non-autophagic mechanism for cell killing.

## Materials and methods

### Cell culture and drug treatment

The colon cancer cell lines HCT116 and HCT116+chromosome 3 (HCT+chr3) cells (Koi *et al*, 1994) were maintained in DMEM with 10% FBS; the latter were cultured with 400 *μ*g ml^−1^ G418 periodically to ensure retention of the extra chromosome 3. The normal (WT) human fibroblast cell line hTERT-1604 (Ouellette *et al*, 2000) with a stably integrated (CA)_17_ microsatellite repeat (Roques *et al*, 2001) and their MLH1-depleted derivatives (M1, M2, etc) were grown in high-glucose DMEM with 10% FBS and 2 × non-essential amino acids, with hygromycin (150 *μ*g *μ*l^−1^) for the siRNA-containing cells. Selection was removed 48 h before any experimental analysis. For MNU (Sigma, Poole, UK) treatment, cells were pretreated with 25 *μ*M 0^6^-benzylguanine (BG; Sigma) to inactivate MGMT and BG was kept in the medium throughout. Other inhibitors were maintained in the media as follows: 3,4-dihydro-5[4-(1-piperindinyl)butoxy]-1(2H)-isoquinoline (DPQ; Sigma) at a final concentration of 30 *μ*M (Yu *et al*, 2002); Boc-D-FMK at 10 *μ*M (Hickman and Samson, 2004); caffeine at 2 mM (Stojic *et al*, 2004) and pifithrin at 20 *μ*M (Zeng *et al*, 2007). Treatment with MNU was for 2 h in all cases: apart from the colony assays, where different concentrations were used, all other experiments used 2 mM MNU. For 6TG (Sigma), medium was changed every 48 h with the drug present throughout. For hydroxyurea (Invitrogen, Paisley, UK), cells were treated at 5 mM for 72 h (Jiang *et al*, 1999).

### Stable RNAi vector assembly and transfection

An siRNA oligonucleotide *MLH1-1733* 5′-AACTGTTCTACCAGATACTCATT-3′ was designed for *MLH1* using an algorithm (Yuan *et al*, 2004; available at http://jura.wi.mit.edu). Overlapping primers incorporating the siRNA sequences were made and ligated into pSilencer (Ambion, Huntingdon, UK) as per the supplier's recommendations. All constructs were verified by sequencing. The vector was linearised (*Sal*I) and 1 × 10^7^ cells electroporated (BioRad GenepulserII; Bio-Rad Laboratories Inc., Hemel Hempstead, UK) with 1 *μ*g of construct in PBS at 320 V and 500 *μ*F. Medium containing serum was immediately added and the cells plated on 90 mm dishes at 5 × 10^5^ cells per dish before selection in hygromycin (150 *μ*g *μ*l^−1^) for 10–14 days.

### Western blotting

Total protein (30 *μ*g) from cells growing in log phase was resolved by SDS–PAGE, electroblotted onto nitrocellulose membranes and blocked for 2 h at room temperature (RT) in 5% non-fat dry milk. Membranes were incubated with anti-MLH1 (BD Pharmingen; G168-15, Oxford, UK), anti-*β*-actin (Abcam; 8226, Cambridge, UK), anti-PMS2 (BD Pharmingen; A16-4), anti-GAPDH (Cell Signaling Technologies; 14C10, Herts, UK), anti-Caspase 7 (Cell Signaling Technologies; 9492), anti-PARP (BD Pharmingen; 4C10-5) or anti-PAR (BD Pharmingen; LP96-10) overnight at 4°C, followed by HRP-conjugated secondary antibody for 2 h at RT before detection using ECL (Amersham Biosciences, Amersham, UK).

### Quantitative PCR and reverse transcriptase–PCR

Total RNA was extracted using the RNeasy kit (Qiagen, Crawley, UK) and cDNA made using oligo d(T)_15_ and reverse transcriptase (Promega, Southampton, UK). The Taqman gene expression assay kit was used for quantitative PCR (QPCR) of *MLH1* (Applied Biosystems, Warrington, UK) with preincubation at 95°C for 10 min, then 40 × 95°C for 15 s and 60°C for 1 min. *MLH1* values were normalised to *GAPDH*. The 2^−ΔΔCT^ method (Bustin, 2000) was used to quantify the fold difference in *MLH1* expression between WT and knockdown and the assay repeated three times. PCR was carried out on cDNA using 1.25 U Taq, 1 × buffer, 3.5 mM MgCl_2_, 0.4 mM dNTPs and 0.5 pmol primer (Invitrogen) at 94°C for 3 min, then 25 × 94°C for 1 min; 60°C for 1 min; 68°C for 1 min and finally 72°C for 10 min. For primer sequences and product sizes see Table 1.

### Cell viability and senescence assays

TUNEL staining was done using the *in situ* Cell Death Detection Kit (Roche, Burgess Hill, UK) following the manufacturer's instructions and counterstaining with DAPI (125 ng *μ*l^−1^). An ethidium bromide/acridine orange staining technique visualised cells which were undergoing late apoptosis or necrosis (Ribble *et al*, 2005), which appear red in both cases. Slides were prepared in duplicate and three independent experiments performed. For senescence associated *β*-galactosidase assays, 1 × 10^5^ cells per well were seeded on six-well plates and allowed to attach overnight, followed by MNU treatment. Number of senescing cells was determined as described (Rambhatla *et al*, 2002); as a positive control the solution was buffered to pH4 to detect non-specific lysosomal *β*-galactosidase activity.

### Cell cycle profile analysis

1 × 10^6^ cells in log phase were harvested, resuspended in 2 ml ice-cold 70% ethanol and stored at −20°C for a minimum of 30 min. They were then centrifuged at 300 **g** for 5 min, resuspended in 400 *μ*l PBS and passed through a 25-gauge needle. To this, 50 *μ*l of 1 mg ml^−1^ RNase and 50 *μ*l of 400 *μ*g ml^−1^ propidium iodide was added. This was incubated for 30 min in the dark at 37°C and placed on ice before counting on a Becton Dickinson (Oxford, UK) FACScalibur II.

### Microsatellite mutation rate analysis

The fluctuation analysis was performed as previously described (Roques *et al*, 2001) with the number of revertants in each subculture corrected for colony-forming efficiency. Mutation rates were calculated using the Luria–Delbruck method of the mean and statistical analysis was as described (Roques *et al*, 2001).

### Clonogenic assays for drug resistance

Cells were seeded at 500 cells per 100 mm dish and after recovery for 24 h were treated with drugs as indicated above. After 14 days, plates were stained with crystal violet and colonies counted; numbers were expressed as a percentage of the colonies formed in the absence of the drug. Assays were carried out in triplicate and three independent experiments were completed.

### Immunofluorescence

Gelatin-coated superfrost slides (BDH, Poole, UK) were sterilised by UV overnight, placed in quadriPERM chamber dishes (Sigma), seeded with 5 × 10^5^ cells and then left overnight again. Drug treatment was as described above. Slides were washed for 3 × 5 min in PBS and fixed for 15 min in 4% ice-cold paraformaldehyde, then washed 3 × 5 min in AB buffer (1% triton X-100, 4% goat serum, 0.2% SDS in PBS). Blocking and permeabilisation was in AB buffer for 3 h before incubation overnight with mouse anti-phospho H2AX antibody (Upstate, Watford, UK) at 1 : 300 dilution, or with rabbit antiapoptosis inducing factor (Cell Signaling Technologies) at 1 : 150 in AB buffer at 4°C. Slides were then rinsed in AB buffer, 3 × 5 min and incubated with goat anti-mouse or anti-rabbit Alexa Fluor 488 (Molecular Probes, Eugene, OR, USA) at 1 : 400 for 1 h at RT, then rinsing as before. Counterstaining was with Hoescht DNA stain (Sigma) before mounting in Vectashield. Images were captured on a Two-photon Laser Scanning Microscope (Leica, Milton Keynes, UK). Experiments were carried out three times; pictures taken are representative of each slide at each time point.

### Statistical analysis

Results, unless noted, are presented as mean±standard deviation (s.d.) for a given number of observations (*n*). Data from each set of observations were cross-compared using unpaired Student's *t*-tests (Graphpad Prism software, GraphPad Software Inc., La Jolla, CA, USA). Differences were considered significant if *P*<0.05.

## Results

### Initial characterisation of fibroblast cell lines depleted in MLH1

**Figure 1:**
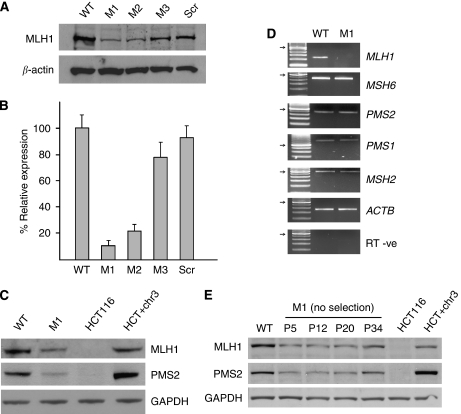
MLH1 depletion in the hTERT-1604 human fibroblast cell line. (**A**) Western blot of total protein from the parental hTERT-1604 cells used for the transfections (WT) and the clonally derived cell lines M1, M2 and M3 each containing a stably integrated MLH1 siRNA vector. Scr denotes cells which contain a scrambled siRNA as negative control. Antibodies used are indicated at left: *β*-actin is a loading control. (**B**) Quantitative PCR (QPCR) showing MLH1 transcription levels in clones compared to WT. Samples were normalised to GAPDH and values represent the mean of each experiment ±2 standard deviations (s.d.). RNA from the cell with the scrambled control (Scr) is the negative control. (**C**) Western blot from the indicated cell lines. The same membrane was probed in succession for each of the antibodies at right. A colon cancer cell line lacking MLH1 (HCT116) and the derived cell line HCT116+chr3 containing MLH1 (HCT+chr3) are shown as controls: GAPDH is a loading control. (**D**) RT–PCR for each of the MMR genes indicated in WT and M1 cells. *β*-Actin (*ACTB*) and a reverse transcriptase negative (RT−ve) sample are positive and negative controls, respectively; the arrow indicates the 500 bp band on the size ladder. (**E**) Western blot of protein extracts from M1 cells grown in the absence of hygromycin (no selection): passage number (P) is indicated. Controls are as in (**C**). The experiments in (**A**–**D**) were carried out at least three times and experiment (**E**) twice.

Human hTERT-1604 fibroblast cells (WT) were transfected with a vector expressing an siRNA specific for *MLH1* and individual resistant colonies picked following growth in hygromycin. Western blotting was used to determine the MLH1 protein levels. Clones varied in the extent of MLH1 depletion, presumably because of insertion site effects. Two clones with low (M1 and M2) and one with intermediate (M3) levels of MLH1 protein were analysed further (Figure 1A), together with cells transcribing a scrambled control (denoted Scr). Real-time PCR was carried out to confirm that the decrease in MLH1 was due to reduced mRNA levels and not an effect on translation and to provide accurate quantitation: levels in M1 (11.6%) and M2 (22.2%) were substantially decreased compared to wild type (Figure 1B), whereas those in M3 cells were intermediate to high (78.5%), with Scr cells (93.30%) essentially wild type (WT).

PMS2 forms the MutL*α* repair complex with MLH1 and requires MLH1 binding for stability (4, 27). M1 cells showed decreased PMS2 levels as seen in the MLH1-deficient cell line HCT116 (Figure 1C). Levels of PMS2 in M2 and M3 clones were comparable to those of MLH1 in those cells (not shown). To ensure that there was no non-specific targeting of PMS2 or other repair components by the siRNA, we carried out reverse transcriptase–PCR (Figure 1D) which shows that transcript levels for *MSH6*, *PMS2*, *PMS1* and *MSH2* were unaffected.

To ensure that the clones identified are indeed depleted in MLH1 because of the presence of the siRNA and not due to picking rare clones with mutations in MLH1 or genes which regulate it, we carried out long-term culturing of M1 cells in the absence of selection for the knockdown construct. This led to a gradual increase in MLH1 levels due a slight growth advantage for cells which have turned off siRNA expression. By passage 34 in the absence of hygromycin, MLH1 levels were significantly higher (Figure 1E), showing that MLH1 depletion can be reversed. This was accompanied by increased PMS2 levels, thus restoring the MutL*α* complex (Figure 1E); these cells were termed M1-R (for ‘rescue’).

### MLH1-deficiency increases cell survival in response to 6TG and MNU

**Figure 2:**
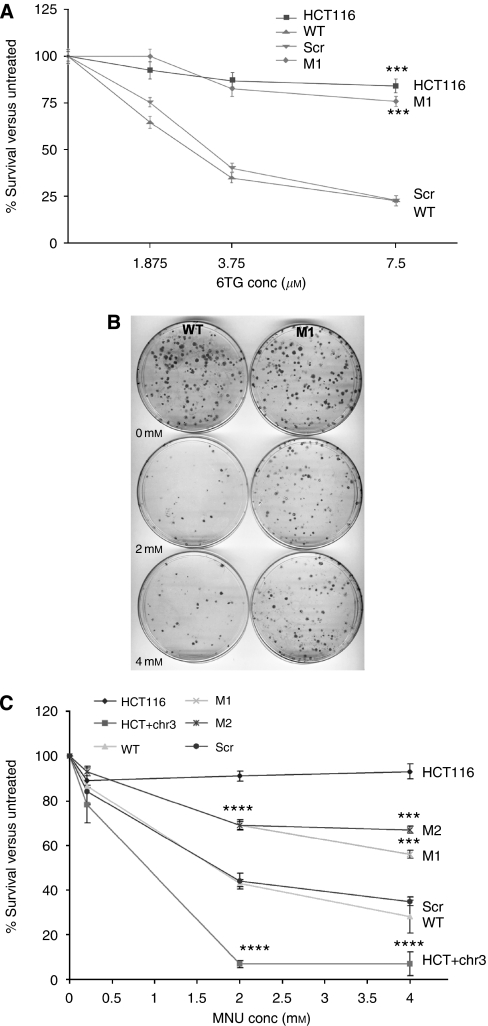
Drug resistance in MLH1-depleted cells. (**A**) Summary of clonogenic assay results in response to 6TG. A total of 500 cells were seeded on each plate and exposed to the indicated concentrations of the drug for 14 days. Plates were stained and the number of colonies formed expressed as a percentage of the number seen on an untreated plate. Values represent the mean of two independent experiments with two plates per cell line: error bars indicate ±s.d. HCT116 is a positive control. M1 and HCT116 survival at 7.5 *μ*M was compared to WT; ^***^P<0.001. (**B**) Examples of clonogenic assay plates for WT (left) and M1 (right) cells following exposure to the indicated levels of MNU. (**C**) Summary of clonogenic assays in response to MNU. HCT116 is a positive control; HCT+chr3 and Scr are negative controls. Statistical comparisons are between matched cell lines (HCT116 *vs* HCT+chr3 or WT *vs* M1 etc). Values represent the mean of three independent experiments, each with three plates per cell line: error bars indicate ±s.d.; ^***^P<0.001; ^****^P<0.0001. M1 and M2 gave similar *P* values at 2 mM MNU. The Scr cells were not significantly different from WT.

Resistance to 6TG is characteristic for cell lines lacking MLH1, and M1 cells were as tolerant to 6TG as HCT116 cells by clonogenic assay (Figure 2A). To test the tolerance of the different MLH1-depleted lines to methylating agents, we exposed the cultures to MNU. WT cells were sensitive to MNU only in the presence of BG, which inhibits the endogenous MGMT activity, confirming that the main cytotoxic lesion being caused by the drug was 0^6^-methylguanine (Hickman and Samson, 2004). Figure 2B shows typical results for MNU treatment in the presence of the inhibitor, clearly illustrating the increased relative survival of the M1 cells. Results for all the cell lines for MNU are summarised in Figure 2C: resistance was similar in M1 and M2 cells, but the scrambled control was indistinguishable from WT. M1-R cells showed similar drug resistance as WT cells (not shown).

Previous studies have indicated that small amounts of MLH1 are enough for normal repair of microsatellites, whereas much higher levels of this protein are required for damage signalling to be functional (Buermeyer *et al*, 1999; Cejka *et al*, 2003). We found the same to be true of our MLH1-depleted cell lines. To test mutation rates, we carried out a Luria–Delbruck fluctuation test (Supplementary Figure 1A) in the M1 cell line, taking advantage of a (CA)_17_ microsatellite reporter with a downstream *neo* gene in the (−1) reading frame which was inserted in the WT cells (Roques *et al*, 2001). The *neo* gene can be brought back in frame by deletion of a single repeat (−2 bp) or insertion of two repeats (+4 bp) in the microsatellite, though larger insertions/deletions are possible. The types of mutations were determined by capillary electrophoresis of fluorescently labelled PCR products (Supplementary Figure 1B). Overall, the microsatellite mutation rates were not significantly different between the M1, WT and Scr cells (Table 2). The relative frequencies of the different type of mutations seen are also summarised in Supplementary Figure 1C and suggest no change in the mutational spectrum in M1 cells. These results confirm that the effects we are seeing in M1 cells are due to the loss of the damage signalling function of MLH1 only, as there is no discernable difference in microsatellite repair function compared to WT cells.

### Absence of initial cell cycle arrest in response to MNU in WT hTERT-immortalised cells

**Figure 3:**
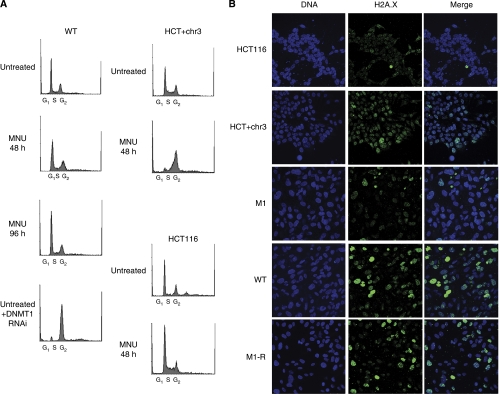
Cell cycle profiles and H2AX signalling in WT and MLH1-depleted cells. (**A**) Cell cycle profiles. Analysis of WT cells which were exposed to 2 mM MNU for 2 h or left untreated, then grown in normal medium, harvested after the indicated time, stained with propidium iodide and analysed by flow cytometry (left). The G_1_, S and G_2_/M fractions are indicated. WT cells arrested in G_2_/M by depletion of DNMT1 (DNMT1 RNAi) are shown as a control. HCT116 and HCT116+chr3 (HCT+chr3) are also shown as controls (right). (**B**) Cells from the cell lines indicated at left were grown on slides and fixed at 48 h after 2 mM MNU treatment before carrying out immunofluorescence with antibody to the phosphorylated form of histone H2AX (green). DNA was counterstained with Hoescht (blue) to localise the nuclei and images captured on a confocal microscope using identical settings: merged images are shown at right. Experiments were carried out at least three times each.

For most mouse fibroblast or human cancer cell lines, exposure to methylating agents leads to G_2_/M arrest after two cell divisions and in an MLH1-dependent manner (Hawn *et al*, 1995; Aquilina *et al*, 1999; Buermeyer *et al*, 1999). WT fibroblasts did not arrest out to 96 h (Figure 3A), however, at doses of MNU which gave significant differences in survival (see Figure 2A and B). We could confirm MLH1-dependent cell cycle arrest by 48 h in HCT116 and HCT116+chr3 (Figure 3A), where the MLH1^+^ cells arrest in the second cell cycle after exposure (Cejka *et al*, 2003; Stojic *et al*, 2004), whereas the MLH1-deficient HCT116 do not. Similar results were found for 6TG: HCT116+chr3 arrested, but WT did not. M1 cells, like WT cells, did not arrest for either drug (not shown). All experiments were repeated at least three times. Telomerase-immortalised fibroblasts have previously been shown to have normal cell cycle arrest in response to various insults (Jiang *et al*, 1999; Vaziri *et al*, 1999; Ouellette *et al*, 2000) and we could confirm that the cells have an intact G_2_/M checkpoint, as they arrest at this point in response to depletion of DNMT1 (Figure 3A; Chen *et al*, 2007). They also show G_1_ accumulation (Supplementary Figure 2A) in response to 5-hydroxyurea (Jiang *et al*, 1999). Some cancer cells can enter senescence with only transient arrest in response to methylating agents (Hirose *et al*, 2001, 2002) and hTERT-immortalised cells can exit the cell cycle and enter senescence in response to stresses such as serum deprivation (Hirose *et al*, 2002; Rambhatla *et al*, 2002), but we found no increase in the number of senescing cells in response to MNU (Supplementary Figure 2B).

### WT cells show a strong MLH1-dependent H2AX phosphorylation response to MNU exposure

Mismatch repair-coupled processing of methylation damage leads to single-strand DNA breaks, which become sites for H2AX phosphorylation (Cejka *et al*, 2003; Mojas *et al*, 2007). This can be seen in HCT116+chr3 cells, which show stronger and more persistent H2AX phosphorylation by 48 h (Figure 3B) than HCT116, as reported previously (Cejka *et al*, 2003; Mojas *et al*, 2007; 12, 24, 48 and 72 h time points also examined; data not shown). WT cells also showed phosphorylation of H2AX at 48 h (Figure 3B), indicating that the cells are indeed detecting the MNU-induced damage in a similar timeframe to the colon cancer cells. Signalling in the WT fibroblasts appeared more widespread and intense than in HCT116+chr3, suggesting a stronger response in these normal cells (experiments were repeated three times). M1 cells showed much lower levels of phosphorylation, indicating that MLH1 depletion prevents normal H2AX signalling in response to MNU in the fibroblasts as well. Confirming that this is due to the absence of MLH1 alone, rescued cells with restored MLH1 levels recovered the ability to strongly activate this damage signal (M1-R; Figure 3B).

### MLH1-dependent cell death involves activation of PARP, but not caspases

**Figure 4:**
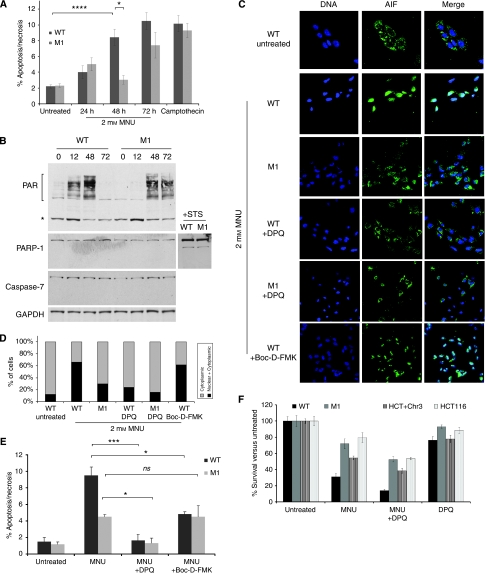
Differences in PARP activity and in viability between WT and MLH1-depleted cells. (**A**) Cell viability was assayed using ethidium bromide/acridine orange staining, which detects both apoptotic and necrotic cells. WT and M1 cells were treated with MNU as indicated and numbers of dying cells counted at the times shown. As a control, cells treated with camptothecin were assayed at 72 h. Values represent the mean of three replicates ±s.d.; ^*^P<0.05; ^****^P<0.0001. The experiment was carried out at least three times. (**B**) WT and M1 cells were treated with 2 mM MNU and protein extracted at the indicated times for western blotting with the antibodies shown at left. The asterisk indicates a background band for this antibody also seen in other studies. GAPDH is shown as a loading control. Extracts from cells 48 h after treatment with staurosporine (STS), which triggers PARP cleavage, are shown for comparison. Experiments were carried out twice. (**C**) WT or M1 cells were seeded onto slides, treated with 2 mM MNU and grown for 48 h before fixing and examining for nuclear or cytoplasmic localisation of AIF (green) by immunofluorescence on a confocal microscope. DNA was counterstained with Hoescht (blue) and merged images are shown at right. DPQ (30 *μ*M) is a PARP inhibitor and Boc-D-FMK (10 *μ*M) a pan-caspase inhibitor. Experiments were carried out three times. (**D**) Quantitative analysis of the effects of MNU and inhibitors on AIF localisation, based on scoring immunofluoresence staining as seen in (**C**) for >100 cells on multiple slides. (**E**) WT and M1 cells were treated with 2 mM MNU, either alone or in combination with 30 *μ*M DPQ or 10 *μ*M Boc-D-FMK, and analysed at 72 h as in (**A**) above; values represent the mean of three samples ±s.d.; ^***^P<0.001; ^*^P<0.05; *ns*, not significant. (**F**) Clonogenic assays in response to 2 mM MNU, 30 *μ*M DPQ or a combination of the two were carried out on the indicated cell lines with two plates per cell line; error bars indicate ±s.d. values. The assay was carried out three times for the fibroblasts (WT and M1), with representative results shown, and once for the controls (HCT116 and HCT116+chr3).

Cell death can occur in the absence of cell cycle arrest under certain circumstances (Peterson-Roth *et al*, 2005; Meador *et al*, 2008). Methylating agents can trigger cell death directly through caspase- (Hickman and Samson, 2004) or PARP-mediated (Yu *et al*, 2002) pathways. Using acridine orange/ethidium bromide staining we found significant differences in viability by 48 h between WT and MLH1-depleted cells treated with 2 mM MNU (Figure 4A). We examined PARP activity over time in the MNU-treated cells by western blotting with an anti-PAR antibody (Figure 4B). Increasing numbers of PAR-labelled proteins became visible from 12 to 48 h in the WT cells, but by 72 h substrate seemed to have been exhausted as almost no PAR groups were detected. In M1 cells however, PAR levels were barely detectable at 12 h, lower than WT at 48 h and were still easily detectable at 72 h (Figure 4B). These results suggested that PARP was being activated in response to the MNU-induced damage and that activation depended in part on MLH1. In contrast, no evidence of caspase activation was seen at these time points: targets of caspase cleavage such as caspase-7 and PARP-1 itself remain uncleaved throughout this period (Figure 4B). PARP-1 cleavage in response to staurosporine, a known initiator of caspase-mediated cell death, is shown for comparison. Likewise, we could detect no evidence for TUNEL staining in WT or MLH1-depleted cells during this period (Table 3).

PARP overactivation can lead to translocation of apoptosis-inducing factor (AIF) from the mitochondrion to the nucleus (Yu *et al*, 2002, 2006). We examined AIF localisation in WT and MLH1-depleted cells (Figure 4C). In untreated WT cells, AIF was present in the cytoplasm in a speckled pattern consistent with its normal mitochondrial localisation; 48 h after treatment with MNU, most AIF can instead be found in the nucleus of the WT cells. In contrast, M1 cells show little or no nuclear staining 48 h after MNU treatment (Figure 4C). To confirm that translocation was dependent on PARP, we treated the WT cells with the PARP inhibitor DPQ as well as MNU: in the presence of DPQ, almost no AIF translocation was seen (Figure 4C). DPQ had no effect on AIF localisation in M1 cells, where it remained cytoplasmic. Substituting a general caspase inhibitor (Boc-D-FMK) instead of DPQ did not block AIF translocation (Figure 4C). Quantitative analysis confirms that MLH1 depletion reduced AIF translocation in response to MNU (Figure 4D) and that translocation is also dependent on PARP, but not caspase activity.

To examine whether inhibiting these events would lead to decreased cell death in response to MNU, we examined cell viability following DPQ treatment. The addition of the PARP inhibitor with MNU led to almost complete abrogation of the short-term effects of MNU on cell viability in both WT and M1 cells (Figure 4E), confirming that MNU initially kills these cells almost exclusively by a PARP-dependent mechanism and that the residual sensitivity to MNU in M1 cells (which may be due to the remaining MLH1) can be overcome by blocking PARP activity. However treatment of cells with DPQ leads to a potentiation of cell killing in response to 2 mM MNU in a clonogenic assay, compared to treatment with MNU alone (Figure 4F), as previously reported. This is normally attributed to the detrimental effects of PARP inhibition in the long term on BER-mediated repair of MNU-induced damage, but may also be partly due to its inherent cytotoxicity, evident in cells treated with DPQ alone (Figure 4F). PARP activation and AIF translocation have been shown to lead to mitochondrial membrane depolarisation, cytochrome c release and subsequent caspase activation (Yu *et al*, 2002). In keeping with this, we found that caspase inhibitors could partly reduce cell death in the WT cells at 72 h (Figure 4E, *P*<0.05). Significantly, caspase inhibitors had no protective effect in MLH1-depleted cells, as levels of cell death were unchanged from those seen in cultures treated with MNU alone, suggesting that in the absence of PARP-mediated AIF translocation, no caspase activation is occurring.

### MLH1, but not ATM/ATR or p53, is required for PARP-mediated cell death in response to MNU

**Figure 5:**
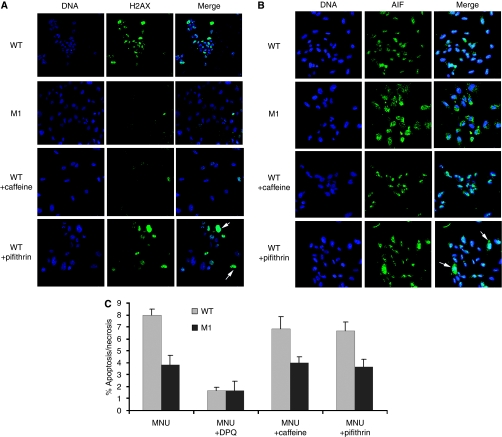
Effects of ATM/ATR and p53 inhibitors on PARP-mediated cell death. (**A**) H2AX phorphorylation (green) was examined using immunofluoresence: nuclei were counterstained with Hoescht (blue). WT cells were treated with 2 mM MNU for 2 h as usual and allowed to recover in the presence or absence of ATM/ATR inhibitor (2 mM caffeine) or p53 inhibitor (20 *μ*M pifithrin). M1 cells are shown for comparison. Images are representative of three independent experiments. Arrows indicate cells showing prominent nuclear staining in the presence of pifithrin. (**B**) AIF localisation was examined using immunofluoresence to AIF (green) and counterstaining for DNA (blue); WT cells were treated with 2 mM MNU and AMT/ATR inhibitor or p53 inhibitor as indicated in (**A**). Images are representative of three independent experiments. Arrows indicate cells showing prominent cytoplasmic staining in the presence of pifithrin. (**C**) Cell viability was assayed following exposure of cells to 2 mM MNU and ATM/ATR inhibitor (caffeine) or p53 inhibitor (pifithrin) as before; values represent the mean of three samples ±s.d. There were no significant differences in viability in the presence of caffeine or pifithrin.

In colon cancer cells, MLH1 requires ATR (Stojic *et al*, 2004) and ATM (Adamson *et al*, 2005) to phosphorylate H2AX and to signal cell cycle arrest, but does not require WT p53 for either process (Cejka *et al*, 2003); however, MLH1 does require p53 to mediate an autophagic response to 6TG (Zeng *et al*, 2007). On treating our WT cells with caffeine, an ATM/ATR inhibitor, we found that little H2AX phosphorylation could be detected (Figure 5A). Pifithrin, a p53 inhibitor, gave some reduction in signal, but not as much as in MLH1-depleted cells (Figure 5A).

To determine if inhibiting ATM/ATR would also prevent AIF translocation, we examined protein localisation in caffeine-treated cells. Using a concentration of inhibitor, which effectively blocked H2AX phosphorylation, we still saw AIF translocate to the nucleus in the majority of cells examined (Figure 5B). Likewise, treatment with the p53 inhibitor failed to block AIF movement in response to MNU in most cells, though a fraction of cells showing cytoplasmic retention of AIF was consistently seen (Figure 5B, arrows). In keeping with these results, treatment of WT cells with caffeine or with pifithrin caused a small but not statistically significant decrease in cell death in response to MNU (Figure 5C). As before, the MLH1-depleted cells showed no change in viability in the presence of the ATM/ATR or p53 inhibitors, suggesting that in the absence of MLH1 these cell death mediators are not substantially activated (Figure 5C).

## Discussion

We show here for the first time that reductions in MLH1 levels cause an impairment of PARP-mediated cell death in response to MNU, implicating MLH1 as an important upstream activator of this caspase-independent cell killing mechanism.

In keeping with previous findings (Buermeyer *et al*, 1999; Cejka *et al*, 2003), low levels of MLH1 were sufficient to allow maintenance of microsatellite stability in our cell lines but insufficient to trigger a normal damage response to MNU or 6TG. The PARP-dependent cell death pathway, like some other MLH1-mediated responses, therefore requires a higher level of MLH1 protein than that needed for DNA repair.

Our results also show that cell death in response to MNU is not necessarily preceded by G_2_/M arrest, because our WT cells show no evidence of arrest well past the point at which significant differences in cell signalling and cell survival are evident between the MLH1-proficient and -depleted cells. Direct progression into a cell death pathway without cell cycle arrest has been noted before under some conditions where DNA damage is extensive (Hirose *et al*, 2002) or H2AX signalling is affected (Meador *et al*, 2008). The marked difference in the strength of H2AX signalling between our WT fibroblasts and the HCT116+chr3 cells is a possible explanation for the difference between these cell lines with regard to cell cycle response. Previous work by a number of labs has shown that cell cycle arrest in HCT116+chr3 is dependent on ATR and ATM (Stojic *et al*, 2004; Adamson *et al*, 2005); in cells lacking these proteins, H2AX phosphorylation is reduced and no arrest occurs in response to MNU. ATM and PARP-1 have also been shown recently to physically and functionally interact following MNNG treatment (Haince *et al*, 2007). We could confirm that ATM/ATR are also important for H2AX phosphorylation in our cells, but that this signal was not required to trigger PARP-mediated cell death, because blocking it could not prevent AIF translocation or increase short-term viability.

The tumour suppressor p53 is a central integrator of stress and cell damage responses and is mutated in almost half of all human cancers. MLH1 triggers an autophagic response to 6TG, but cannot do so if p53 is inactivated (Zeng *et al*, 2007). However, neither the cell cycle arrest (Cejka *et al*, 2003) nor the caspase-mediated apoptosis responses (Hickman and Samson, 1999), which MLH1 can invoke in some cells in response to MNU, appear to be p53-dependent. We found that blocking p53 gave some reduction in H2AX phosphorylation in response to MNU, but failed to prevent AIF translocation or cell death in the majority of cells. These results are consistent with other reports suggesting that the PARP-mediated cell death pathway may be partially independent of p53 (Zong *et al*, 2004; Moubarak *et al*, 2007).

Differences in H2AX phosphorylation between the WT and MLH1-depleted cells are seen 48 h after MNU treatment, as for HCT116 and HCT116+chr3 cells (Cejka *et al*, 2003; Stojic *et al*, 2004), and are likely to be developing after the second round of DNA replication. This is the same time point at which PARP activity has peaked in WT fibroblasts and where differences in viability are detectable between WT and MLH1-depleted cells. By 72 h, PARP activity has fallen to zero in WT cells, suggesting NAD+ depletion, which is known to trigger AIF release from the mitochondrion (Yu *et al*, 2002; Zong *et al*, 2004; Moubarak *et al*, 2007). PARP activity in the MLH1-depleted cells is first evident at 48 h at a lower intensity than in WT cells. Consistent with this, we see far higher AIF translocation to the nucleus in WT cells compared to the MLH1-depleted cells at this time. Up to this point, no evidence of caspase activation can be seen: no protein cleavage, TUNEL staining or sub-G_1_ fraction on FACS analysis were detected. AIF translocation has been shown to lead to later cytochrome c release and caspase activation (Yu *et al*, 2002; Moubarak *et al*, 2007). Our results are also consistent with some late activation of caspase-mediated cell death, but only in cells where MLH1 is at WT levels and can first activate PARP. In a previous study, Samson and colleagues found that although MLH1 was required to activate caspase cleavage in cells treated with a methylating agent, cell death still occurs even in the presence of caspase inhibitors or *bcl-2* overexpression (Hickman and Samson, 2004). The data we present here suggest that PARP-mediated AIF translocation is a candidate for the alternative cell death mechanism triggered by methylating agents.

Although the PARP inhibitor DPQ decreased cell death in response to MNU in the short term, it augmented the cell killing effect of MNU in longer-term clonogenic assays. Both of these effects have been well documented before (Tentori *et al*, 1999; Yu *et al*, 2002; Curtin *et al*, 2004; Zong *et al*, 2004). This apparent paradox can be resolved by considering the types of damage induced by MNU and the two distinct responses, repair or cell death, which can be triggered by PARP in the cell. MNU generates a number of damaged bases in addition to 0^6^-methylguanine, mostly *N*-methylpurines, but these latter are promptly dealt with by the base excision repair (BER) system. PARP facilitates BER by catalysing the addition of PAR groups to histones, opening the chromatin for BER access. If levels of damage are too high, PARP becomes overactive and this depletes the cell of NAD, the substrate for PAR, leading to mitochondrial membrane depolarisation and eventual cell death (Yu *et al*, 2002; Zong *et al*, 2004). Inhibition of PARP thus can block the early overactivation of the enzyme and NAD depletion, leading to short-term gains in cell viability, but at a cost to BER, the failure of which leads to the independent triggering of cell death in the longer term by adducts such as *N*^3^-methyladenine (Engelward *et al*, 1998). Thus, combining PARP inhibitors with MNU can help to kill MLH1-deficient cells, which are resistant to 0^6^-methylguanine, by instead making them sensitive to the *N*-methylpurine damage. We did not see preferential killing of MLH1-deficient *vs* MLH1-proficient colon cancer cells (HCT116) or fibroblasts (M1) using DPQ and MNU, as has been reported using temozolomide and second-generation PARP inhibitors such as AG14361 (Curtin *et al*, 2004) or NU1025 (Tentori *et al*, 1999). These newer PARP inhibitors have lower cytotoxicity and higher specificity than DPQ, which has non-specific cytotoxic effects including interference with nucleoside metabolism (Milam *et al*, 1986) and we show here that it has a detrimental effect on long-term cell survival in the absence of MNU.

MMR deficiency is an important clinical correlate of drug resistance. The use of methylating agents such as temozolomide can give rise to MMR-defective tumours following treatment of ovarian (Brown *et al*, 1997) and brain tumours (Cahill *et al*, 2007) and is an independent predictor of poor prognosis after chemotherapy in breast (Mackay *et al*, 2000) and oesophageal (Kishi *et al*, 2003) cancer patients. Our data indicate that MLH1-deficient cells have a survival advantage against PARP-dependent cell killing through AIF, as well as against caspase-mediated cell death, which was previously known (Hickman and Samson, 1999, 2004). Examining the signalling pathway between MLH1 and PARP may help in the design of strategies for overcoming chemotherapeutic resistance caused by MMR defects. As epigenetic silencing of the *MLH1* promoter is commonly seen in tumours showing acquired resistance (Herman and Baylin, 2003), our work also suggests that combining strategies designed to reactivate the silenced *MLH1* (such as treatment with DAC; Plumb *et al*, 2000) with S_N_1 methylating agents may promote PARP-mediated cell death. This independent cell death pathway may be particularly useful in cells where the caspase-mediated route has been blocked due to microsatellite mutations at BAX or ATR (Bacon *et al*, 2001; Mongiat-Artus *et al*, 2006). As MLH1 deficiency is sufficient to confer resistance to a range of other clinically relevant chemotherapeutic agents, including cisplatin and 6TG, our model system will be of great use in determining whether MLH1 is involved in signalling PARP in response to these agents as well.

In conclusion, our results show that MLH1 is required to induce a caspase-independent cell death mechanism in response to the S_N_1-type methylating agent MNU. This mechanism is independent of the cell cycle response, ATM/ATR or p53 but is dependent on PARP activation and subsequent AIF translocation.

## Figures and Tables

**Table 1:** Primer sequences and PCR product sizes for RT–PCR

**Gene**	**Primers**	**Product size (bp)**
*MLH1*	F: 5′-TGGGACGAAGAAAAGGAATG -3′	250
	R: 5′-GATCAGGCAGGTTAGCAAGC-3′	
*MSH6*	F: 5′-GCACGAGTGGAACAGACTG-3′	430
	R: 5′-CGGGTATCAGACCTTCCTG-3′	
*PMS1*	F: 5′-CACTTCGGTGGTCAGTGTTG-3′	713
	R: 5′-GATGAAACTTCTTTCTGGTGTTG-3′	
*PMS2*	F: 5′-CCCATGGTAGAAAAGCAGC-3′	533
	R: 5′-GCGAGATTAAGTTGGCTGAG-3′	
*MSH2*	F: 5′-GCTGGAAATAAGGCATCCA-3′	441
	R: 5′-CATCTGCTCTCCCTTTTTG-3′	
*ACTB*	F: 5′-AACTGGAACGGTGAAGGTG-3′	350
	R: 5′-TCAAGTTGGGGACAAAAG-3′	

**Table 2:** Rate of mutation of reporter (CA)_17_ repeat in MLH1-depleted cells (M1) and controls

**Cell line**	**Mean cloning efficiency**	**Mean G418 resistant colonies per plate**	**Proportion of colonies with microsatellite frameshifts**	**Microsatellite mutation rate (mutations per cell per generation)**
	0.51	5	1	1.11 × 10^−5^
WT	0.52	9	1	**1.56 × 10^−5^**
	0.48	11	0.81	1.78 × 10^−5^
	0.39	20	0.77	6.64 × 10^−6^
M1	0.49	14	0.95	**1.78 × 10^−5^**
	0.48	16	1	5.63 × 10^−5^
	0.5	4	1	9.2 × 10^−6^
Scr	0.56	9	1	**1.15 × 10^−5^**
	0.51	6	0.9	1.4 × 10^−5^

For mutation rate analysis, the colony forming efficiency with respect to unselected controls, number of resistant clones, and the fraction of clones with a frameshift in the microsatellite region were determined. Each line represents the respective value for three independent experiments, with median values for the mutation rates indicated in bold. (ie, three replicates × three independent experiments for WT gave an overall median mutation rate for all nine samples of 1.56 × 10^−5^). The difference between the median values for the three groups is not statistically significant (M1 *vs* WT, *P*=0.4705; Scr *vs* WT, *P*=0.2468).

**Table 3:** Percentage of WT and MLH1-depleted (M1) human fibroblasts undergoing apoptosis in the presence of MNU

	**% Apoptotic cells (±s.e.m.)**
DNase treated positive control (WT)	97.90 (±0.9)
	
*WT*	
Untreated	1.51 (±0.5)
2 mM MNU 12 h	2.50 (±0.9)
2 mM MNU 24 h	1.48 (±0.5)
	
*M1*	
Untreated	1.51 (±0.5)
2 mM MNU 12 h	1.58 (±0.4)
2 mM MNU 24 h	1.6 (±0.8)

Cells were seeded on poly-L-lysine slides and apoptosis measured by the TUNEL method. Values shown are the mean percentage±s.e.m. of duplicate slides, counting cells in 10 fields at × 20 magnification. Three independent experiments were conducted. The positive control was WT cells treated with DNaseI prior to the addition of the terminal deoxynucleotidyl transferase.
