# MLH1 mediates PARP-dependent cell death in response to the methylating agent *N*-methyl-*N*-nitrosourea

**DOI:** 10.1038/sj.bjc.6605615

**Published:** 2010-03-16

**Authors:** J R McDaid, J Loughery, P Dunne, J C Boyer, C S Downes, R A Farber, C P Walsh

**Correction to:** *British Journal of Cancer* (2009) **101**, 441–451; doi:10.1038/sj.bjc.6605186

Owing to an error during the final correction of this paper, panels E and F in Figure 4 were incorrectly typeset, resulting in the loss of part of panel 4E. The publishers apologise for this mistake.

The correct panels 4E and 4F are reproduced below:

## Figures and Tables

**Figure 4:**
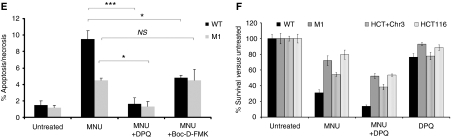
Differences in PARP activity and in viability between WT and MLH1-depleted cells. (**E**) WT and M1 cells were treated with 2 mM MNU, either alone or in combination with 30 mM DPQ or 10 mM Boc-D-FMK; as a control, cells treated with camptothecin were assayed at 72 h; values represent the mean of three samples ±s.d.; ^***^P<0.001; ^*^P<0.05; NS, not significant. (**F**) Clonogenic assays in response to 2 mM MNU, 30 mM DPQ or a combination of the two were carried out on the indicated cell lines with two plates per cell line; error bars indicate ±s.d. values. The assay was carried out three times for the fibroblasts (WT and M1), with representative results shown, and once for the controls (HCT116 and HCT116þchr3).

