# Supplementary figures and images for: MLH1 mediates PARP-dependent cell death in response to the methylating agent N-methyl-N-nitrosourea
Source: Br J Cancer. 2009 Jul 21;101(3):441–51. doi: 10.1038/sj.bjc.6605186 (PMC2720233; doi:10.1038/sj.bjc.6605186)

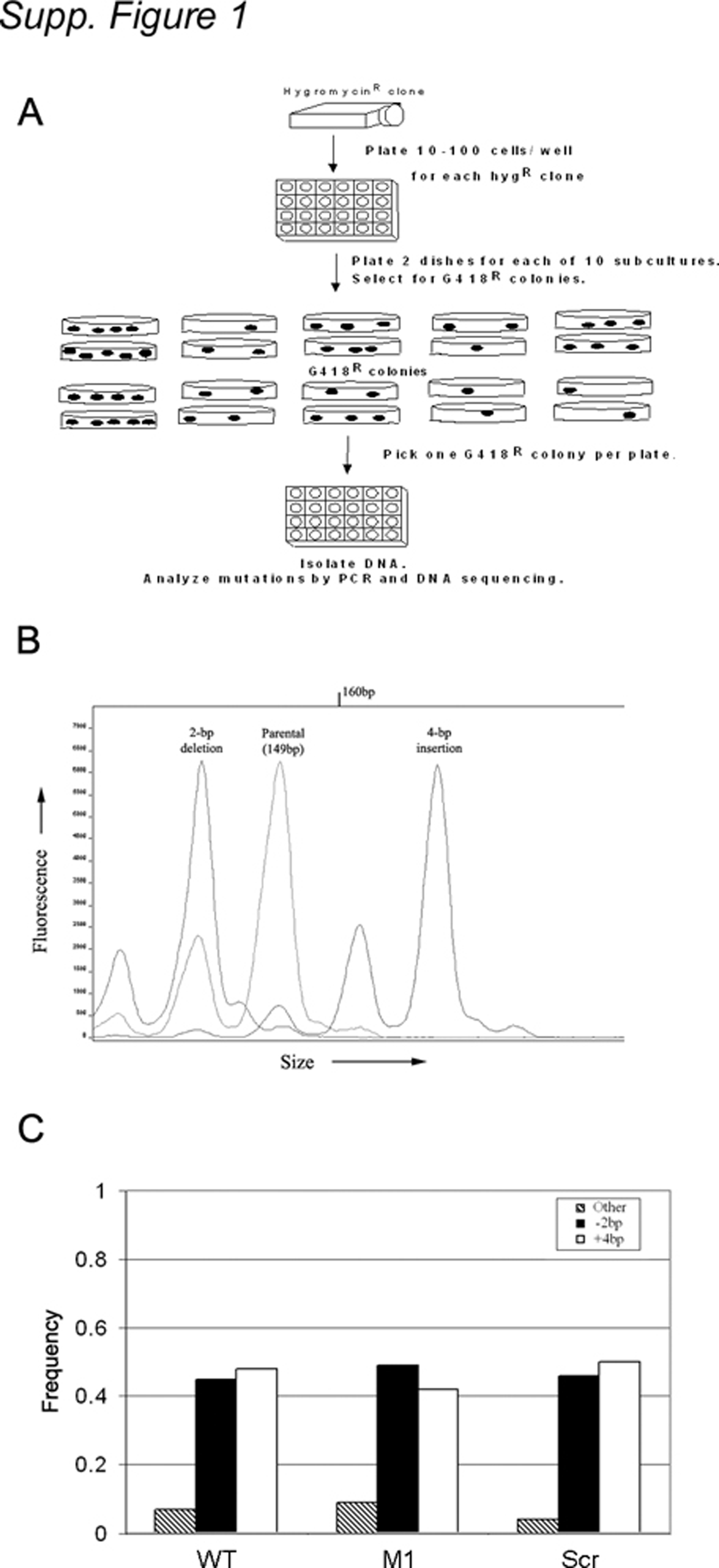

Supplement: Supplementary Figure 1 [file 6605186x1.tif]

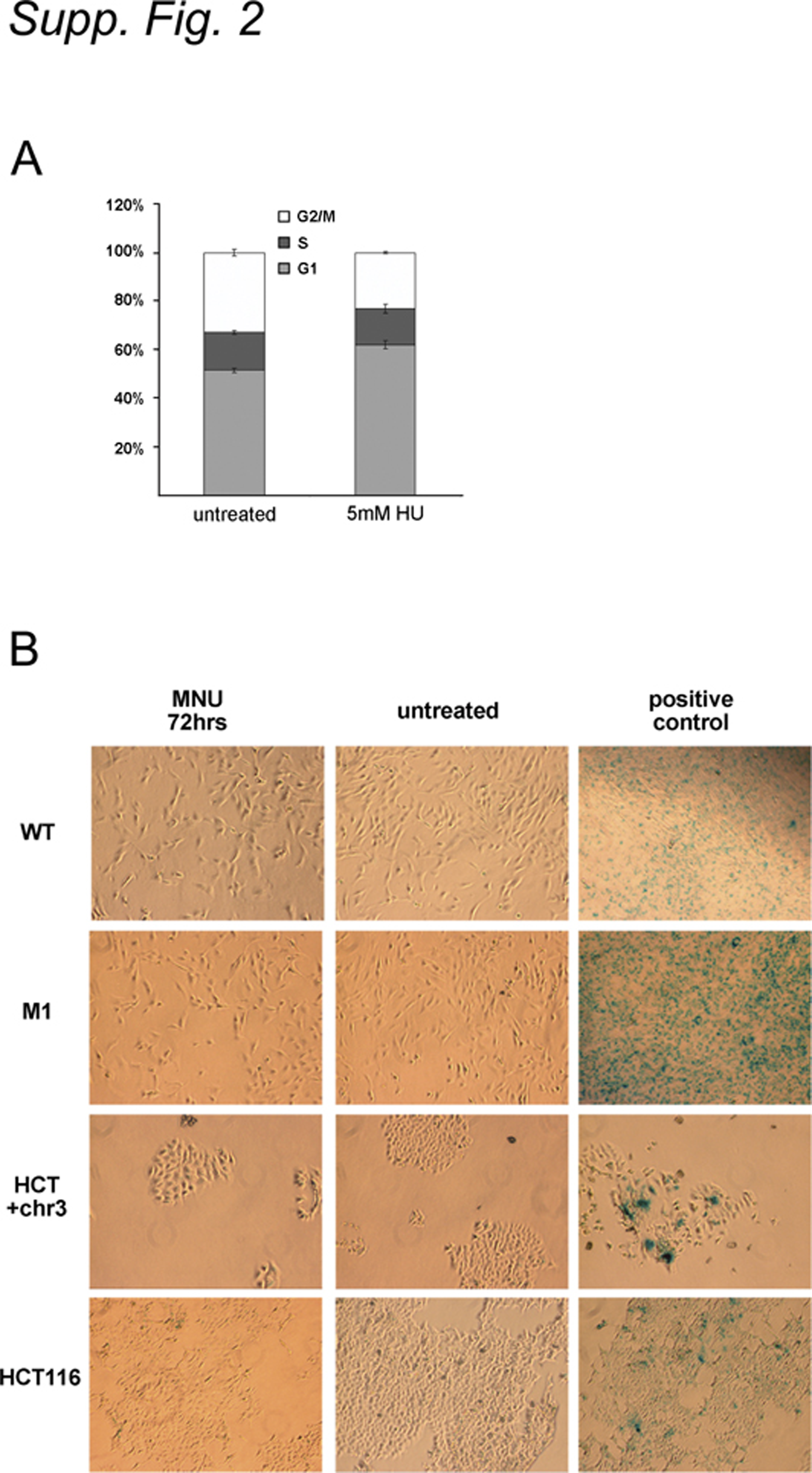

Supplement: Supplementary Figure 2 [file 6605186x2.tif]
