# Supplementary material for: MLH1 mediates PARP-dependent cell death in response to the methylating agent N-methyl-N-nitrosourea
Source: Br J Cancer. 2009 Jul 21;101(3):441–51. doi: 10.1038/sj.bjc.6605186 (PMC2720233; doi:10.1038/sj.bjc.6605186)
Supplement: Supplementary Figure Legends [file 6605186x3.doc]

*Supplementary Figure 1. Microsatellite mutation rates in MLH1-depleted cells.*

*A.* Schematic outlining the Luria-Delbruck fluctuation test. Cells carrying the siRNA construct are hyrogromycin-resistant. These were grown further in the presence of G418 and individual *neo^R^* colonies counted. *B.* DNA was extracted from sample colonies and PCR with one fluorescently-labelled primer carried out over the microsatellite region in the reporter. Products were analysed on the ABI sequencer using GENESCAN software and a size standard (160bp). Examples of mutations (a 2-bp deletion and a 4-bp insertion) which put the *neo^R^* gene back in frame are shown. *C.* Relative frequencies of insertions and deletions for the indicated cell lines.

*Supplementary Figure 2. G1 checkpoint and senescence in WT cells.*

*A.* WT fibroblasts were treated with 5mM hydroxyurea (HU) for 72hrs, harvested, fixed and stained with propidium iodide before analyzing by flow cytometry. The fraction of cells in each stage of the cell cycle is indicated: values indicate the mean of three samples +/- SEM. The experiment was carried out with duplicate samples at least three times. *B.* Cells from the indicated cell lines were seeded onto six-well plates, treated with 2mM MNU for 2hrs and allowed to recover for 72hrs, then fixed and stained for senescence-associated β-galactosidase (blue). Untreated cells are shown as a negative control and cells stained for non-specific β-galactosidase shown as a positive control. The experiment was carried out in triplicate and representative fields are shown.
